# The Factors Associated With COVID-19 Infection Among Healthcare Workers at a Tertiary Care Institution in North India: A Case-Control Study

**DOI:** 10.7759/cureus.52475

**Published:** 2024-01-18

**Authors:** Ravneet Kaur, Shashi Kant, Mohammad Ahmad, Arvind Kumar, Suneeta Meena, Mohan Bairwa, Rakesh Kumar, Anisur Rahman

**Affiliations:** 1 Centre for Community Medicine, All India Institute of Medical Sciences, New Delhi, IND; 2 NPO Research, World Health Organization, New Delhi, IND; 3 Medicine, All India Institute of Medical Sciences, New Delhi, IND; 4 Laboratory Medicine, All India Institute of Medical Sciences, New Delhi, IND; 5 Health and Research Emergency Office, World Health Organization, New Delhi, IND

**Keywords:** hand hygeine, infection prevention and control, personal protective equipment, health-care workers, covid-19

## Abstract

Background and objective

Healthcare workers (HCWs) are at a higher risk of contracting coronavirus disease 2019 (COVID-19) since they regularly come into direct contact with infected patients and their surroundings. In light of this, it is critical to study the potential risk factors for SARS-CoV-2 infection among HCWs to help determine its transmission patterns and prevent infections among HCWs, as well as healthcare-associated COVID-19.

Methods

We conducted a case-control study at a tertiary healthcare center from December 2020 to August 2021. HCWs who tested positive for severe acute respiratory syndrome coronavirus 2 (SARS-CoV-2), the virus that causes COVID-19, by RT-PCR were included as cases and those who tested negative for RT-PCR and SARS-CoV-2 antibodies were recruited as controls. We interviewed 316 HCWs (187 cases and 129 controls) by using the WHO questionnaire titled “Protocol for assessment of potential risk factors for coronavirus disease 2019 among health workers in a health care setting” to assess infection prevention and control (IPC) knowledge and practices, including the use of personal protective equipment (PPE). The odds ratio (OR) for factors associated with infection was determined by multivariable logistic regression.

Results

The majority (87.2%) of the cases were symptomatic. Adherence to IPC measures was higher among controls as compared to cases. A significantly higher number of controls used PPE compared to cases. The proportions of HCWs involved in cleaning, patient transport, reception, and catering were higher among cases (37.9%) compared to controls (19.1%). In multivariable analysis, undergoing training on care for COVID-19 patients was associated with a lower risk of infection (OR: 0.40, 95% CI: 0.24-0.69).

Conclusions

Adherence to IPC and use of PPE were significantly higher among controls as compared to cases. Receiving training in COVID-19 patient care and compliance with IPC measures were associated with a lower risk of COVID-19 infection among HCWs in this study.

## Introduction

The first case of coronavirus disease 2019 (COVID-19) in India was detected on January 30, 2020. Thereafter, the country witnessed two major waves in the years 2020 and 2021. In July 2020 alone, more than one million cases and 25,000 deaths were reported. The cumulative number of reported cases was more than 32 million, with four million deaths till August 2021 [1,2]. As the pandemic progressed, healthcare workers (HCWs) were increasingly exposed to COVID-19 patients. HCWs are at a higher risk of infection as they come into regular direct contact with patients and their surroundings that may be contaminated. In addition, HCWs also pose a risk of transmitting the infection to other HCWs or the members of their household [3].

The transmission of severe acute respiratory syndrome coronavirus 2 (SARS-CoV-2), the virus that causes COVID-19, to HCWs in healthcare settings weakens the healthcare delivery systems. HCWs provide clinical care to patients and are responsible for the implementation of infection prevention and control (IPC) measures, including the optimal use of personal protective equipment (PPE), that play a critical role in reducing transmission of infection in healthcare settings [3]. Hence, it is crucial to study the potential risk factors for SARS-CoV-2 infection among HCWs, as it will help prevent the healthcare-associated spread of COVID-19 in healthcare settings.

To this end, we adapted the WHO questionnaire titled “Protocol for assessment of potential risk factors for coronavirus disease 2019 among health workers in a health care setting” [4] and conducted this study at our hospital, as a part of the WHO multi-center case-control study. The objective of our study was to describe the range of clinical presentations and assess the risk factors of SARS-CoV-2 infection in HCWs.

## Materials and methods

Study design: case-control study

Study Setting

This study was conducted at a tertiary healthcare center in northern India. The institute has 1,700 beds and includes comprehensive facilities for patient care, research, and medical teaching. Along with the main hospital, there are specialty centers for cardiothoracic and neurosciences, ophthalmology, cancer, trauma center, drug dependence treatment center, and a rural hospital with 50 beds. There are 49 departments (clinical and paraclinical) involved in patient care services. The institution has designated areas for COVID-19 care, an out-patient screening facility, an in-patient facility for quarantine, and admissions, and a critical care facility, as well as non-COVID-19 areas. The designated virology laboratory of the institute for testing COVID-19 has been approved by the Indian Council of Medical Research [5]. 

Sample Size

Considering a two-sided confidence level of 95%, power of study of 80%, the ratio of controls to cases as 2, the hypothetical proportion of controls with exposure as 40, and assumed odds ratio (OR) of COVID-19 infection for exposure of 1.70, and by factoring in a 10% non-response rate, the sample size was estimated to be 200 cases and 400 controls. When the study was planned, no sufficient evidence was available on the risk factors; hence the sample size was calculated based on the assumed OR of potential exposures [6].

Study Participants

The study was conducted among HCWs working at the study institution. The institution had a total of 17,650 employees, comprising 3,150 doctors, approximately 5,000 nurses, 2,700 technicians, 1,800 security personnel, about 2,000 administrative staff, and nearly 3,000 contractual employees working as hospital attendants, as well as sanitation workers [5]. 

Operational Definitions

Healthcare worker (HCW): Any employee of the healthcare facility involved in the provision of care to a COVID‑19 patient, including those who may not be directly involved in patient care activities, but who had contact with the patient’s body fluids, potentially contaminated items or environmental surfaces, such as cleaning and laundry personnel, X-ray technicians, administrative staff, phlebotomists, respiratory therapists, nutritionists, social workers, physical therapists, laboratory personnel, cleaners, admission/reception clerks, patient transporters, and catering staff.

Cases: A case was an HCW who was exposed in a healthcare setting to a COVID-19 patient in the 14 days before testing positive for SARS-CoV-2 RT-PCR.

Controls: A control was defined as an HCW who was exposed in a healthcare setting to a COVID-19 patient in the 14 days before recruitment and had a negative RT-PCR for SARS-CoV-2 and a negative antibody serology test for SARS-CoV-2 within the first week (days one to seven) of the positive RT-PCR test.

Exposure: Contact with a patient having laboratory-confirmed COVID-19 infection, including exposure to the patient’s blood and body fluids, and to contaminated materials or devices and equipment linked to the patient.

Recruitment of Cases and Controls

The existing contact-tracing mechanism in the study institute was used for the identification of cases and controls. Whenever an HCW was reportedly infected with SARS-CoV-2, a doctor-in-charge at the respective department contacted the Central Contact Tracing Team (CCTT). The HCW could also directly report to CCTT by calling the central helpline telephone number. The details of the contact tracing mechanism are described in another study [7].

Enrolment of Cases

Once an HCW was identified as RT-PCR-positive for COVID-19, made aware of the study requirements, and consent was obtained to be included, the HCW was enrolled as a case, after fulfilling the inclusion criteria. The cases were contacted by phone, study procedures were explained, and informed consent for interview and blood collection was sought at this time. For those who consented, the interview was conducted over the telephone and an appointment was fixed for blood specimen collection. The blood specimen was collected either at home or the hospital (in case the HCW was admitted), within seven days of the positive RT-PCR test. 

Enrolment of Controls

Controls were selected from the Employees Health Services (EHS) screening clinic, where HCWs exposed to COVID-19 cases reported for RT-PCR testing. Based on exposure, the HCWs were stratified into high- and low-risk groups.

High-risk exposure was defined as follows:

(i) Performed respiratory aerosol-generating procedures (AGPs) without any one of the following protection measures: N95 face mask, eye/face protection, and gloves.

(ii) If the patient’s body fluids, respiratory tract secretions, or saliva came into contact with non-intact skin or mucous membrane.

(ii) Present within 1 meter of the confirmed case without a mask (both case and contact) for more than 15 minutes.

All other exposures were considered as low-risk exposures. 

As part of the standard operating procedure of the institute, all HCWs with a history of high-risk exposure to a confirmed COVID-19 patient were tested after five days of exposure. HCWs with low-risk exposure but having symptoms suggestive of COVID-19 infection were also tested [7]. The HCWs who were exposed to COVID-19 patients, but tested negative on RT-PCR as well as negative for SARS-CoV-2 antibodies, tested within one week of RT-PCR positive result, were recruited as controls. Data were collected during the period from December 2020 to August 2021.

The HCWs were interviewed using the WHO questionnaire “Protocol for assessment of potential risk factors for coronavirus disease 2019 among health workers in a health care setting” [4]. Information regarding demographic details, symptoms, use of medication, availability and adherence to IPC measures, and contact with and exposure to COVID-19 patients was collected. 

Testing for SARS-CoV-2 antibodies

The serological testing for COVID-19 IgG antibodies was done with the kits provided by the WHO (Wantai ELISA kits). The ELISA for total antibodies detection was developed based on a double-antigen sandwich-based immunoassay (Ab-ELISA), using recombinant SARS-CoV-2 antigen [8]. The testing was performed at the Department of Laboratory Medicine, All India Institute of Medical Sciences, New Delhi as per the manufacturer’s (Beijing Wantai Biological Pharmacy Enterprise Co. Ltd.) instructions. 

Quality assurance

The laboratory had both internal and external quality control procedures in place. All the equipment involved in the testing was routinely calibrated. The laboratory used LIS (laboratory information system) for reporting results, which minimized post-analytical errors.

Ethical considerations

Informed consent was obtained from all the study participants. The results of the tests were communicated. Confidentiality was maintained and the dataset was password-protected. The study was conducted after obtaining ethical approval from the Institute Ethics Committee of the All India Institute of Medical Sciences, New Delhi. (Ref. No. IEC-997/03.10.2020). The manuscript was approved by the Publication Review Committee of the World Health Organization.

Statistical analysis

Data were entered into Epi Info version 6 and analyzed using IBM SPSS Statistics v 25 (IBM Corp., Armonk, NY). The categorical variables were expressed as numbers and percentages (95% CI). Cases and controls were compared across variables, using a chi-square or Fisher's exact test for categorical variables, and a t-test for continuous variables. Bi-variate analysis, followed by a step-wise multiple regression analysis, was done to identify the factors significantly associated with SARS-CoV-2 infection among the study population. The level of significance was set at 0.05 and 95% confidence intervals were estimated.

## Results

**Figure 1:**
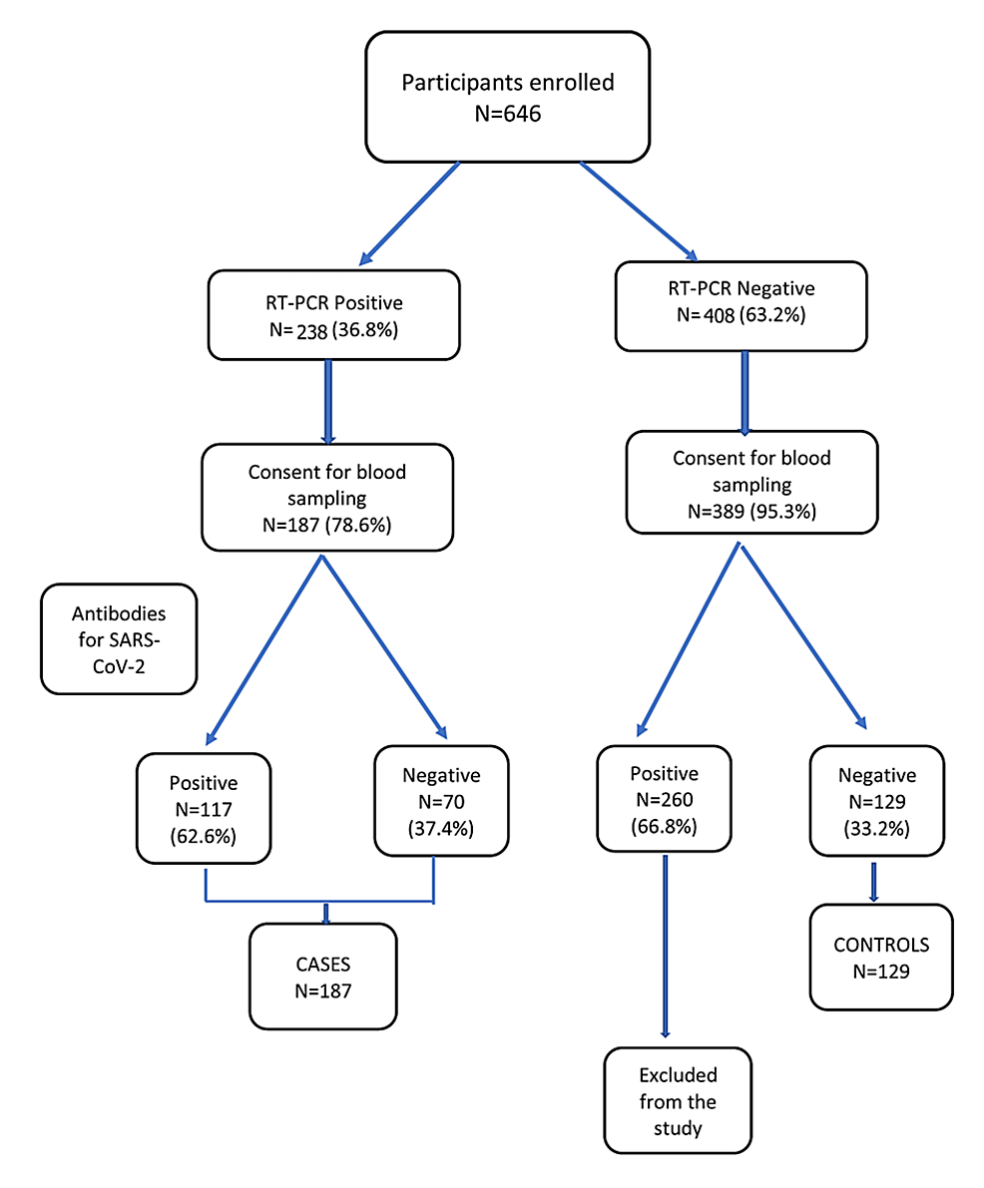
Flow diagram depicting the selection of participants for the study SARS-CoV-2: severe acute respiratory syndrome coronavirus 2

We enrolled a total of 646 participants initially Of these, 238 (36.8%) were RT-PCR-positive and were included as cases. Out of 238 cases, 187 (78.6%) consented to provide blood samples, and 389 of 408 (95.3%) RT-PCR-negative participants consented to give blood samples as well. Of these 389 participants, 260 (66.8%) were seropositive for SARS-CoV-2 antibodies and hence excluded from the study. Thus, we included 129 participants as controls, i.e., those who had tested negative for both RT-PCR and SARS-CoV-2 antibodies (Figure 1).

The sociodemographic and clinical characteristics of the participants are presented in Table 1. The proportion of female participants was similar in cases (46%) and controls (48.8%). Nurses and technicians constituted 45.5% of the cases and 52.7% of the controls. The proportions of HCWs involved in cleaning, patient transport, reception, and catering were higher among cases (37.9%) compared to controls (19.1%). However, the proportion of doctors was significantly lower among cases (16.6%) compared to controls (27.9%; p=0.003).

**Table 1:** Distribution of study participants by demographics and other selected characteristics HCW: healthcare worker

Characteristic		Cases	Controls	P-value
		N (%)	N (%)	
Sex	Female	86 (46.0)	63 (48.8)	0.618
	Male	101 (54.0)	66 (51.2)	
Years of education, years	5-10	17 (9.1)	12 (9.3)	0.949
	More than 10	170 (90.9)	117 (90.7)	
Occupation	Medical doctor	31 (16.6)	36 (27.9)	0.003
	HCWs involved in direct patient care, other than doctors	85 (45.5)	68 (52.7)	
	HCWs involved in cleaning and patient transport	24 (12.8)	13 (10.1)	
	HCWs not involved in direct patient care (reception clerks, food bearers, administrative staff)	47(25.1)	12 (9.3)	
Symptom status	Symptomatic	163 (87.2)	35 (27.1)	<0.001
	Asymptomatic	24 (12.8)	94 (72.9)	
Total		187 (100)	129 (100)	

In our study, 87.2% of the cases were symptomatic. Fever (71.8%) and cough (69.9%) were the most common symptoms among the cases, followed by muscle aches (62.6%), headaches (62%), fatigue (58.3%), runny nose (45.4%), and loss of smell (40.5%). Other common symptoms were joint aches, loss of appetite, nausea, and diarrhea. Shortness of breath was reported in 6.7% of the cases (Table 2).

**Table 2:** Distribution of clinical symptoms among cases (n=163) Multiple responses possible; 24 RT-PCR-positive HCWs were asymptomatic HCW: healthcare worker; RT-PCR: reverse transcription–polymerase chain reaction

Symptom	Number	%
Fever	117	71.8
Cough	114	69.9
Muscle ache	102	62.6
Headache	101	62
Fatigue	95	58.3
Runny nose	74	45.4
Loss of smell	66	40.5
Joint ache	62	38
Loss of appetite	51	31.3
Diarrhea	34	20.9
Nausea	24	14.7
Chills	23	14.1
Shortness of breath	11	6.7
Vomiting	9	5.5
Nosebleed	4	2.5
Seizures	4	2.5
Conjunctivitis	3	1.8
Rash	1	0.6
Altered consciousness	1	0.6

As shown in Table 3, the majority of the HCWs (100% of the cases and 96.9% of the controls) had received training in IPC. About 63% of the cases and 78% of the controls reported that they always followed IPC measures during patient care, while 23% of the cases and 10.9% of the controls reported following the IPC measures occasionally. The difference was statistically significant (p=0.08; OR: 0.46, 95% CI: 0.28-0.77). A significantly higher proportion of controls (80%) compared to cases (66%) were aware of the importance of hand hygiene. The majority of the cases (73.8%) and controls (79.8%) reported that they performed the recommended hand hygiene. However, a significantly higher proportion of controls compared to cases reported performing hand hygiene before touching a patient (82.2% vs. 69%; p=0.006, OR: 0.48, 95% CI: 0.28-0.83), before a procedure (80.6% vs. 59.9%; p<0.001, OR: 0.36, 95% CI: 0.21-0.60), after exposure to body fluids (78.3% vs. 61%; p<0.001, OR: 0.43, 95% CI: 0.26-0.72), and after touching the patients’ surroundings (83.7% vs. 72.2%; p=0.02 OR: 0.51, 95% CI: 0.28-0.88).

**Table 3:** Distribution of cases and controls based on adherence to IPC practices CI: confidence interval; COVID-19: coronavirus disease 2019; IPC: infection prevention and control; OR: odds ratio; PPE: personal protective equipment

Variables		Cases (n=187)	%	Control (n=129)	%	P-value	OR (95% CI)
Specific training in COVID-19 patient care	No	144	77.0	67	51.9	0.000	3.09 (1.91–5.03)
	Yes	43	23.0	62	48.1		
Received IPC training	No	0	0.0	4	3.1	0.013	0.0 (0.0–0.75)
	Yes	187	100.0	125	96.9		
Mode of PPE training	Both virtual and physical	26	13.9	48	37.2	0.000	0.26 (0.15–0.45)
	Either virtual or physical	161	86.1	77	59.7		
	Not aware of PPE training	0	0.0	4	3.1		
Hand hygiene technique	Partially known/not known	63	33.7	26	20.2	0.009	2.01 (1.19–3.40)
	Known	124	66.3	103	79.8		
Frequency of hand hygiene	Always, as recommended	138	73.8	103	79.8	0.14	0.63 (0.35–1.16)
	Most of the time	40	21.4	19	14.7		
	Occasionally	9	4.8	7	5.4		
Hand hygiene before touching the patient	Always, as recommended	129	69.0	106	82.2	0.022	0.48 (0.28–0.83)
	Most of the time	39	20.9	13	10.1		
	Occasionally	19	10.2	10	7.8		
Hand hygiene before a procedure	Always, as recommended	112	59.9	104	80.6	0.000	0.36 (0.21–0.60)
	Most of the time	19	10.2	7	5.4		
	Occasionally	56	29.9	18	14.0		
Hand hygiene after exposure to body fluids	Always, as recommended	114	61.0	101	78.3	0.000	0.43 (0.26–0.72)
	Most of the time	11	5.9	13	10.1		
	occasionally	62	33.2	15	11.6		
Hand hygiene after touching the patients	Always, as recommended	129	69.0	109	84.5	0.006	0.41 (0.23–0.72)
	Most of the time	37	19.8	11	8.5		
	Occasionally	21	11.2	9	7.0		
Hand hygiene after touching the patient's surroundings	Always, as recommended	135	72.2	108	83.7	0.021	0.51 (0.28–0.88)
	Most of the time	41	21.9	13	10.1		
	Occasionally	11	5.9	8	6.2		
Followed IPC practices when in contact with the patients	Always, as recommended	117	62.6	101	78.3	0.008	0.46 (0.28–0.77)
	Most of the time	43	23.0	14	10.9		
	Occasionally	27	14.4	14	10.9		
Use of PPE	Appropriate	127	67.9	110	85.3	0.002	0.36 (0.21–0.65)
	Inappropriate	60	32.1	19	14.7		
Total		187	100	129	100		

Adherence to the appropriate use of PPE was significantly higher among the controls (85.3%) compared to cases (67.9%) (p=0.002). In multivariable analysis (adjusted for sex, age, occupation, IPC training, use of PPE, and hand hygiene), undergoing COVID-19-specific training was associated with a lower risk of acquiring COVID-19 infection (OR: 0.4; 95% CI: 0.24-0.69) (Table 4).

**Table 4:** Multivariable logistic regression analysis of factors associated with COVID-19 infection in HCWs *Nurses, technicians (operation theatre, laboratory, radiodiagnosis), dieticians, physical therapists CI: confidence interval; COVID-19: coronavirus disease 2019; HCW: healthcare worker; OR: odds ratio; PPE: personal protective equipment

Variable	Category	Cases, n (%), (n=187)	Controls, n (%), (n=129)	Unadjusted OR (95% CI)	P-value	Adjusted OR (95% CI)	P-value
Sex	Female	86 (46.0)	63 (48.8)	Ref		Ref	
	Male	101 (54.0)	66 (51.2)	1.12 (0.72–1.78)	0.618	1.14 (0.67–1.94)	0.632
Occupation	Doctors	31 (16.6)	36 (27.9)	Ref			
	HCWs involved in direct patient care, other than doctors*	85 (45.5)	68 (52.7)	0.69 (0.39–1.23)	0.205	0.83 (0.42–1.62)	0.576
	HCWs involved in cleaning and patient transport	24 (12.8)	13 (10.1)	0.47 (0.20–1.07)	0.071	0.77 (0.27–2.21)	0.627
	HCWs not involved in direct patient care (reception clerks, food bearers, administrative staff)	47 (25.1)	12 (9.3)	0.20 (0.06–0.67)	0.009	0.39 (0.10–1.58)	0.189
Specific training in COVID-19 patient care	No	144 (77.0)	67 (51.9)	Ref			
	Yes	43 (23.0)	62 (48.1)	0.32 (0.20–0.52)	<0.001	0.40 (0.24–0.69)	0.001
Appropriate use of PPE	No	60 (32.1)	19 (14.7)	Ref			
	Yes	127 (67.9)	110 (85.3)	0.37 (0.21–0.65)	0.001	0.59 (0.25–1.42)	0.241
Appropriate hand hygiene technique	No	63 (33.7)	26 (20.2)	Ref			
	Yes	124 (66.3)	103 (79.8)	0.50 (0.29–0.84)	0.009	1.13 (0.49–2.65)	0.772

## Discussion

In the present study, we have reported the clinical presentations and assessed the factors associated with SARS-CoV-2 infection among HCWs in a tertiary care health facility. In our study, nearly 87% of the cases were symptomatic. This finding aligns with that of another study conducted at a COVID-19-care hospital in Mumbai, where 85% of the HCWs with COVID-19 infection were symptomatic [9]. However, Chandran et al. (2022), in their study conducted in Kerela, in southern India, reported a lower proportion (67%) of symptomatic cases among SARS-CoV-2-positive HCWs [10]. In a study conducted in Italy, Magnavita et al. (2020) reported that nearly 70% of HCWs manifested symptoms of COVID-19 infection [11]. This may be due to differences in the number of symptoms included for screening, as well as the use of a different method to collect the information (online questionnaire through e-mail as opposed to face-to-face/telephonic interview as in our study). As reported in other studies conducted in India and abroad, fever, myalgia, and cough were the predominant symptoms [12-15].

Bivariate analysis revealed that the cases included a significantly higher proportion of HCWs involved in cleaning and patient transport, catering staff, and reception clerks compared to controls. However, this was not found to be significant in the multivariate analysis. It is possible that due to the small number of study participants in various sub-categories of occupation, we might have missed the statistical association even if it existed. In another study conducted in Delhi, Dev et al. (2021) reported that the cases included a significantly higher proportion of sanitation workers (24% vs. 8%) and technical staff (10% vs. 4%) but a lower proportion of doctors (23% vs. 43%) when compared to controls [13]. This, however, contrasts with the findings of a study conducted in Italy that reported the highest rate of infection among physicians compared to other HCWs [14]. This may be due to the difference in adherence to IPC measures, training status, and exposure to patients and their surroundings. In a previous study conducted at the current study site, it was reported that informal workplace interactions (having meals together) and inconsistent use of masks during routine work (39.6%) were the most common causes of high-risk exposure among HCWs, as compared to the patient care activities without appropriate PPE (20.4%) [10]. In a prospective study involving 1,344 HCWs in Paris, Contejean et al. (2020) also reported that major reasons for SARS-CoV-2 infection were contact without PPE with an index case, and taking off masks during breaks in the presence of colleagues [15]. 

Most of the HCWs in our study (100% of cases and 96.5% of controls) were trained in IPC measures. In a study conducted at a tertiary care hospital in Chandigarh (northern India), Sharma et al. (2021) also reported that the majority of the HCWs were trained in IPC [16]. We found that adherence to IPC measures, performing hand hygiene as recommended, and appropriate use of PPE during patient care were associated with a lower risk of COVID-19 infection. This is in line with Dev et al. who reported similar findings [13]. Ran et al. (2020), in their study conducted in Wuhan, China, also reported that non-adherence to recommended hand hygiene practices and inappropriate use of PPE were associated with increased risk of SARS-CoV-2 infection among HCWs [17].

The strengths of our study include the collection of data within seven days after the RT-PCR test, which minimized the recall bias. The inclusion of cases was based on positive RT-PCR tests, while controls were included based on negative serology for SARS-CoV-2 antibodies. The tests were conducted at a laboratory with strict internal and external quality assurance mechanisms. 

The study has a few limitations. The adherence to hand hygiene practices, IPC measures, and the use of PPE were self-reported. Therefore, social desirability bias could not be completely ruled out. A higher proportion of RT-PCR-negative controls (66.8%) had to be excluded from the study due to pre-existing antibodies against SARS-CoV-2. Therefore, the originally planned case-control ratio of 1:2 could not be achieved, which may have reduced the power of the study. Hence, for some variables, we might have missed the statistical association even if it truly existed. However, our study corroborates the existing evidence on the importance of IPC training and the appropriate use of PPE in minimizing the risk of acquiring SARS-CoV-2 infection among HCWs.

## Conclusions

In our study, HCWs involved in cleaning, patient transport, reception, and catering had higher odds of having COVID-19 infection as compared to nurses and doctors. The majority of the cases were symptomatic. Adherence to appropriate use of PPE was significantly higher among the controls (85.3%) compared to cases. Undergoing training in COVID-19 patient care and adherence to IPC measures were associated with a lower risk of COVID-19 infection among HCWs.
